# Long-Term Persistence of Robust Antibody and Cytotoxic T Cell Responses in Recovered Patients Infected with SARS Coronavirus

**DOI:** 10.1371/journal.pone.0000024

**Published:** 2006-12-20

**Authors:** Taisheng Li, Jing Xie, Yuxian He, Hongwei Fan, Laurence Baril, Zhifeng Qiu, Yang Han, Wenbing Xu, Weihong Zhang, Hui You, Yanling Zuo, Qing Fang, Jian Yu, Zhiwei Chen, Linqi Zhang

**Affiliations:** 1 Department of Infectious Disease, Peking Union Medical College Hospital and AIDS Research Center, Chinese Academy of Medical Sciences and Peking Union Medical College Beijing, China; 2 Institute of Pathogen Biology, Chinese Academy of Medical Sciences and Peking Union Medical College Beijing, China; 3 Lindsley F. Kimball Research Institute, New York Blood Center New York, New York, United States of America; 4 Emerging Diseases Epidemiology Unit, Institute Pasteur Paris, France; 5 Aaron Diamond AIDS Research Center, The Rockefeller University New York, New York, United States of America; New York University School of Medicine, United States of America

## Abstract

Most of the individuals infected with SARS coronavirus (SARS-CoV) spontaneously recovered without clinical intervention. However, the immunological correlates associated with patients' recovery are currently unknown. In this report, we have sequentially monitored 30 recovered patients over a two-year period to characterize temporal changes in SARS-CoV-specific antibody responses as well as cytotoxic T cell (CTL) responses. We have found persistence of robust antibody and CTL responses in all of the study subjects throughout the study period, with a moderate decline one year after the onset of symptoms. We have also identified two potential major CTL epitopes in N proteins based on ELISPOT analysis of pooled peptides. However, despite the potent immune responses and clinical recovery, peripheral lymphocyte counts in the recovered patients have not yet been restored to normal levels. In summary, our study has, for the first time, characterized the temporal and dynamic changes of humoral and CTL responses in the natural history of SARS-recovered individuals, and strongly supports the notion that high and sustainable levels of immune responses correlate strongly with the disease outcome. Our findings have direct implications for future design and development of effective therapeutic agents and vaccines against SARS-CoV infection.

## Introduction

SARS, or severe acute respiratory syndrome, is a serious respiratory illness caused by a novel variant of coronavirus (SARS-associated coronavirus, SARS-CoV) [1]–[8]. Others and we have previously demonstrated that the persistent and high levels of N protein-specific and S glycoprotein-specific neutralizing antibody (Nab) responses during the first several weeks of infection are correlated with the disease outcomes [9]–[15]. However, little is known about magnitude and longevity of the both humoral and CTL responses after prolonged recovery. Studying the long-term changes in humoral and CTL responses in recovered patients will not only verify the earlier findings from short-term follow-ups but also to further establish correlates of protection to be generated by future vaccine candidates.

## Results

Starting in March 2003, we have enrolled and sequentially followed up 30 patients who were diagnosed and recovered from SARS-CoV infection according to clinical criteria released by the World Health Organization (http://www.who.int/csr/sars/casedefinition/en). Sequential blood samples were collected at 1, 3, 6, 12 and 24 months after the onset of symptoms from the enrolled patients at the Department of Infectious Diseases, Peking Union Medical College Hospital in Beijing under the guidelines of the ethical review committee at Hospital. Recovered patients were defined as those free from the acute illness (high body temperature, dry cough or light-white sputum, shortness of breath, hypoxia, and air-space consolidation in lungs) approximately 1 month after the onset of symptom with definitive sero-positivity against SARS-CoV lysates at least two consecutive occasions. Clinically, these recovered patients regain their normal body temperature, experience no cough or chest pain, and have normal chest radiograph and normal pulmonary function. The average age of these patients were 37±11 with 13 are being male and 17 female. All the participating patients were antibody and antigen negative for HIV-1, cytomegalovirus (CMV), and Epstein-Barr virus (EBV). For purposes of comparison, blood samples were also obtained from 70 normal healthy age matched individuals. The average age for these individuals is 39±10 with 36 are being male and 34 female.

**Figure 1:**
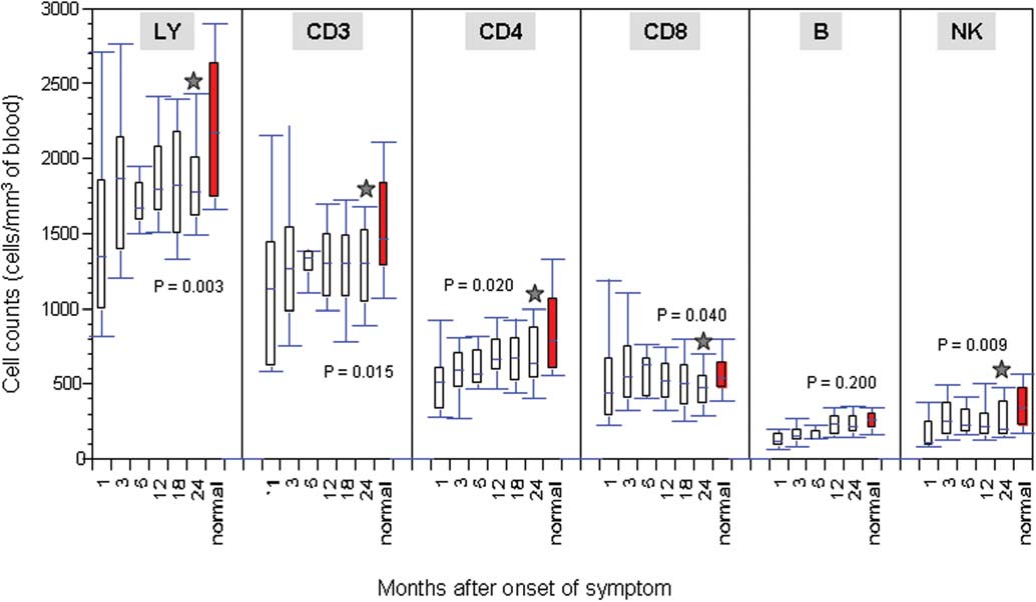
Sequential analysis of peripheral changes in total lymphocytes (LY), CD3, CD4, CD8, B cell, and NK cell counts in recovered patients over 2-year period after onset of symptom. Grey stars indicate the significant differences between the recovered patients at 24 months after onset of symptom and normal healthy controls. Their respective *p* values are presented.

Using flow cytometry, we first studied the sequential changes in the absolute numbers of total lymphocytes, CD3, CD4, CD8 T lymphocytes, B lymphocytes and natural killer (NK) cells over the two years follow-ups and compared with that from normal healthy controls. As show in Fig. 1, recovered patients clearly experienced two distinct phases of cell restoration in the peripheral blood; an initial rapid phase for all the cell populations studied in the first 3 months after the onset of symptoms followed by a significant slower phase during the subsequent months. During the first 3 months, the average increase for the cell populations studied was as high as 22% per month. The mean absolute total lymphocytes, CD3, CD4, and CD8 T lymphocytes, B lymphocytes and NK cells has increased from 1349 to 1870 cells/mm^3^, 1130 to 1268 cells/mm^3^, 511 to 591 cells/mm^3^, 440 to 547 cells/mm^3^, 120 to 152 cells/mm^3^, and 103 to 254 cells/mm^3^, respectively. The rapid phase for lymphocyte recovery is reminescinet of what had reported through the cross-sectional studies on the recovered SARS patients during the first few weeks of onset of symptom [2], [7], [8], [16], [17]. As we and others shown previously, the initial rapid phase in peripheral lymphocyte recovery usually coincided with the improving clinical condition of SARS patients [2], [7], [8], [16], [17]. After the first 3 months, however, the percent of increase dropped to 0.07% per month and, in most cases, remained unchanged or slightly decreased from the previous time points (Fig. 1). Such distinct rate of lymphocyte recovery in the two phases is likely reflective of different mechanisms in lymphocyte regeneration, proliferation and distribution *in vivo*. Furthermore, with the exception for B lymphocytes, the mean absolute numbers for total lymphocytes, CD3, CD4, CD8 T lymphocytes, and NK cells at 24 months after the onset of symptom remained statistically different from that in normal healthy age-matched controls. This finding suggests that complete restoration of peripheral lymphocyte may require a longer period or peripheral lymphocyte reduction in SARS patients is permanent despite of recovery from clinical manifestation of SARS-CoV infection. Longer follow-ups of these patients will be needed to address these two possibilities.

**Figure 2:**
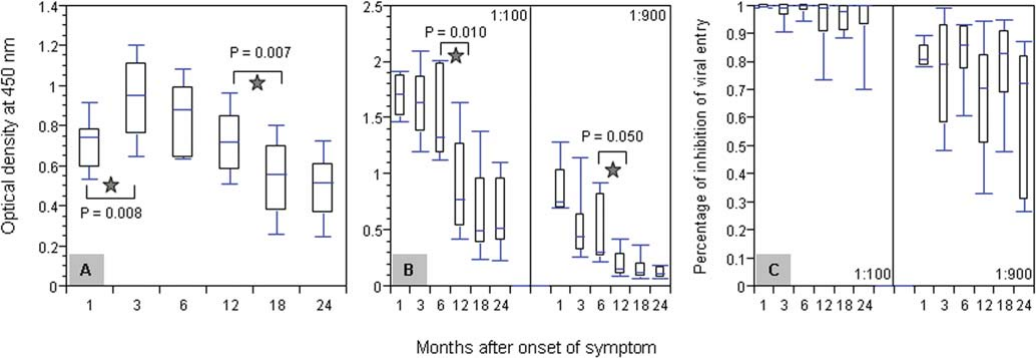
Sequential analysis of antibody responses against the whole viral lysates (A), N protein (B), and pseudotyped virus with S protein (C) of SARS-CoV in 30 recovered patients over 2-year period. Serum samples were diluted 100- and 900-fold prior to ELISA and neutralization studies. Both dilutions were used to carried out experiments presented in (B) and (C), where the only 100-fold diluted serum were used for experiments presented in (A). The top and bottom of each rectangular box denote the 75^th^ and 25^th^ percentiles, respectively, with the median shown inside the box. Horizontal bars extending from each box represent the 90^th^ and 10^th^ percentiles. Significant differences between samples are indicated by grey stars with their respective *p* values. Otherwise no significance was found.

To study the sequential changes in humoral responses against SARS-CoV, we used our previously published ELISA-based and pseudotyped retrovirus-based neutralization systems [11]. We first analyzed the temporal changes in total serum IgG specific for SARS-CoV, using a commercially available ELISA assay (No S20030004, HuaDa Comp, Beijing, China) based on purified whole virus lysates. As shown in Fig. 2A, there was an initial increase in total IgG from month 1 to 3 after onset of symptom followed by a gradual decrease over the ensuing period. In fact, much of the decrease was observed during month 3 to 18 after onset of symptom with no significant changes were found afterwards (Fig. 2A). In addition, we also characterized temporal changes in N protein-specific antibody and S glycoprotein-specific neutralizing antibody (Nab) responses in these patients. Our experiments were conducted with serum samples in two different dilutions (1/100 and 1/900) and Fig. 2B depicts the temporal changes in N protein-specific antibodies over the 24-month follow-up based on N protein-based ELISA. We have found that antibodies against N protein were detectable throughout the entire period of study. Within the first 6 month after onset of symptom, N protein-specific antibody remained relatively high although there is a clear trend of decrease over time, with more significant drop in titers between month 6 and 12 after onset of symptom and no dramatic changes afterwards (Fig. 2B). The same trend of changes in N protein-specific antibodies was also observed for experiments conducted with 1/900 dilution (Fig. 2B). In regard to S glycoprotein-specific Nab, we used our previously published pseudotyped retroviral system with S glycoprotein on the surface of virion and HIV-1 proteins encapsulated within [11]. Consistent with what have been observed for N-protein specific antibodies, high and sustainable levels of S glycoprotein-specific Nab were detected throughout the entire phase of study (Fig. 2C). At 1/100 dilution, all samples had potent neutralizing activities capable of neutralizing at least 60% of input virion, with median level activities larger than 95% (Fig. 2C). There is also of trend of decrease in S glycoprotein-specific Nab titer over time, but no dramatic drop was found between month 6 and 12 as was found for the N protein-specific antibodies. Lastly, the same trend of changes in S glycoprotein-specific Nab was also observed for experiments conducted with 1/900 dilution (Fig. 2C). It remains to be seen whether the levels of SARS-CoV-specific antibodies will remain unchanged or continuously decline after 24 months of follow-up.

**Figure 3:**
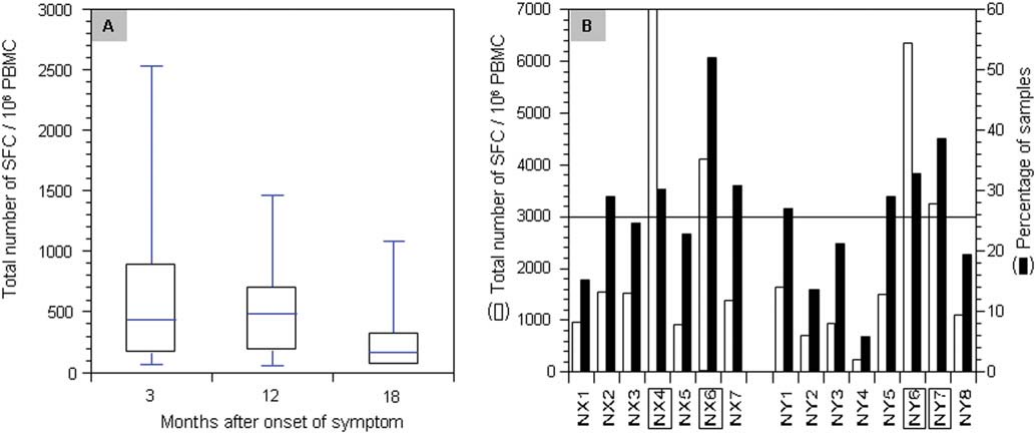
Sequential analysis of CTL responses against a pool of peptides derived from N protein in 30 recovered patients over 2-year period. (A) The average number of spot forming cells (SFC) per million of PBMC from recovered patients at month 3, 12, and 18 post onset of symptom. No significant differences were found among these samples. The top and bottom of each rectangular box denote the 75^th^ and 25^th^ percentiles, respectively, with the median shown inside the box. Horizontal bars extending from each box represent the 90^th^ and 10^th^ percentiles. (B) The average number of SFC per million of PBMC against various pools of N protein peptides (open rectangle) and percent of PBMC samples recognizing various pools of N protein peptides (closed rectangle). An arbitrary line was draw at 3000 SFC per million PBMC to identify peptide pools that are preferentially recognized by the recovered patients in this cohort.

**Figure 4:**
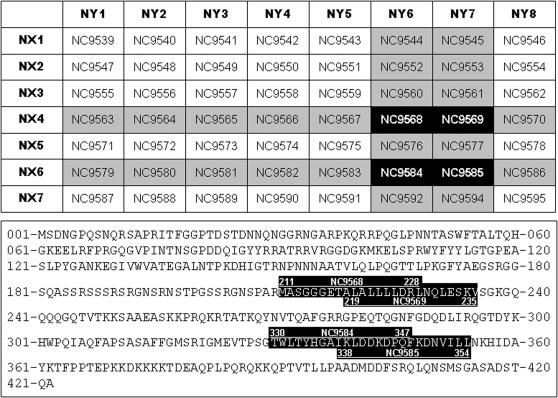
The actual composition and cross-broad layout of each peptide pools derived from the N protein (upper panel). The 57 peptides (one is not listed) were pooled into 15 groups, 7 of which (NX1-7) are listed vertically and 8 are (NY1-8) listed horizontally. The actual amino acid residue sequences preferentially recognized by the recovered patients are highlighted (lower panel), which correspond to residue sequences between position number 211 to 235 and 330 to 354.

To study the sequential changes in CTL responses against SARS-CoV, we used ELISPOT-based technique to quantify the number of INF-γ releasing cells in the peripheral blood against peptide pools covering the entire N protein derived from the Urbani strain [3]. The peptide pools were made of 57 peptides which are 15–18 amino acid residues in length overlapping by 10 amino acid residues. The peptide pools were obtained through the Biodefense and Emerging Infections Resources Repository at National Institute of Health (NIH) in the United States (http://www.beiresources.org/listing/index.cfm). We first studied the sequential changes in total number of INF-γ releasing cells, expressed as Spot Forming Cell (SFC) per million PBMC, in the peripheral blood over the 18 months after onset of symptom. Fig. 3A shows the average number of INF-γ releasing cells detected at month 3, 12 and 18 after onset of symptom. As observed for antibody responses, detectable levels of CTL responses were found throughout the study period, although there is a clear trend of decrease over time (Fig. 3A). To further identify the major epitopes recognized by the CTL, the 57 peptides were further divided into 15 peptide pools in a cross-broad fashion (NX1-7 vs. NY1-8) (Fig. 4, upper panel). The intensity and magnitude of CTL responses against the NX1-7 and NY1-8 peptide pools were presented as either the total number of SFC per million PBMC or the percent of samples having detectable levels of INF-γ releasing cells (Fig. 3B). Fig. 3B summarizes results for all the samples tested and four peptide pools, namely NX4, NX6, NY6, and NY7, were found to be preferentially recognized by recovered SARS patients (Fig. 3B). Through the cross-broad analysis, we have found that there are potential two major CTL epitopes in the N protein. One is located within the region covered by peptides NC9568 and NC9569, and the other is covered by peptides NC9584 and NC9585 (Fig. 4, upper panel). Peptides NC9568 and NC9569 correspond to amino acid sequence MASGGGETALALLLLDRLNQLESKV between positions 211 and 235 in the N protein, whereas peptides NC9584 and NC9585 match the sequence of TWLTYHGAIKLDDKDPQFKDNVILL between positions 330 and 354 (Fig. 4, lower panel). Serotyping of MHC class I and II have found that A2, 3, 11 and 24, B51 and 60, and DR4, 9, 12, and 15, and DQ5, 6, 7, 8, and 9 are the predominant among our study population (Table 1). Future work would be required to further fine mapping the actual CTL epitopes in these two regions and their association with particular serotypes of MHC class I.

**Table 1:**
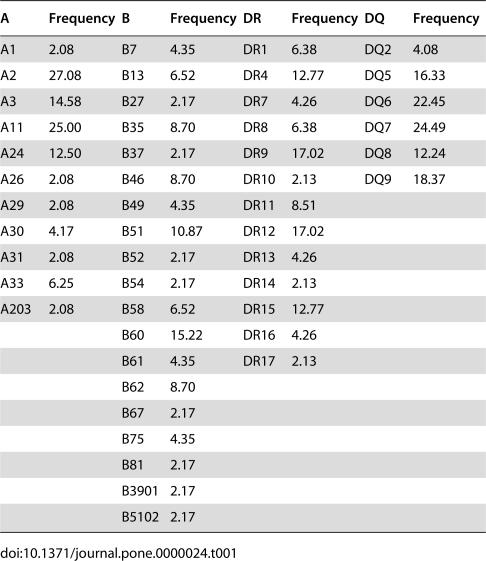
MHC class I and II alleles and their frequency among the study population

A	Frequency	B	Frequency	DR	Frequency	DQ	Frequency
A1	2.08	B7	4.35	DR1	6.38	DQ2	4.08
A2	27.08	B13	6.52	DR4	12.77	DQ5	16.33
A3	14.58	B27	2.17	DR7	4.26	DQ6	22.45
A11	25.00	B35	8.70	DR8	6.38	DQ7	24.49
A24	12.50	B37	2.17	DR9	17.02	DQ8	12.24
A26	2.08	B46	8.70	DR10	2.13	DQ9	18.37
A29	2.08	B49	4.35	DR11	8.51		
A30	4.17	B51	10.87	DR12	17.02		
A31	2.08	B52	2.17	DR13	4.26		
A33	6.25	B54	2.17	DR14	2.13		
A203	2.08	B58	6.52	DR15	12.77		
		B60	15.22	DR16	4.26		
		B61	4.35	DR17	2.13		
		B62	8.70				
		B67	2.17				
		B75	4.35				
		B81	2.17				
		B3901	2.17				
		B5102	2.17				

## Discussion

In this report, we have extended our early study by sequentially monitoring 30 recovered SARS patients over a 2-year period to characterize temporal changes in humoral and CTL responses against SARS-CoV. We have shown for the first time that recovered patients have persistent and robust binding as well as neutralizing antibody and CTL responses throughout the study period with a moderate decline one year after the onset of symptoms. In particular, S glycoprotein-specific Nab responses are persistently high in the recovered patients even 24 months after onset of symptom. Such high and sustainable levels of SARS-CoV-specific immune responses in these patients are clear distinction from that found in patients who succumbed to the disease [11], suggesting that antibody responses likely play an important role in determining the ultimate disease outcome of SARS-CoV infected patients. In addition, we have also identified two potential major CTL epitopes in N protein based on ELISPOT analysis of 57 peptides. Future work will be required to identify the actual epitopes in greater details. However, despite the potent immune responses and clinical recovery, peripheral lymphocyte counts in the recovered patients have not yet been restored to normal levels. This finding suggest that complete restoration of peripheral lymphocyte may require longer period of time, or the lymphocyte destruction during SARS-CoV infection is permanent and can only be repaired partially. In summary, our study has for the first time characterized the temporal and dynamic changes of humoral and CTL responses in the natural history of SARS-recovered individuals and strongly support the notion that high and sustainable levels of immune responses correlated strongly with the disease outcome. We strongly believe that our findings have direct implications for the design and development of therapeutics and vaccines against SARS-CoV infection and replication.

## Materials and Methods

### Study subjects

Thirty SARS patients were enrolled in March 2003 and have continuously followed up since then. They were diagnosed and recovered from SARS-CoV infection according to clinical criteria released by the World Health Organization (http://www.who.int/csr/sars/casedefinition/en). Sequential blood samples were collected with their informed consent and approval from the ethical review committee at the Department of Infectious Diseases, Peking Union Medical College Hospital in Beijing. The average age of these patients were 37±11 with 13 are being male and 17 female. For purposes of comparison, blood samples were also obtained from 70 normal healthy age matched individuals. The average age for these individuals is 39±10 with 36 are being male and 34 female.

### Flow cytometry

For flow cytometric analyses of various lymphocyte populations in the peripheral blood, we used our previously published protocols [17].

### Antibody responses detected by ELISA and pseudotyped retrovirus

Analysis of binding antibodies against whole SARS-CoV lysates or N protein, and S glycoprotein-specific neutralizing antibody (Nab) were conducted as previously reported [11].

### ELISPOT assay

ELISPOT assays were performed using a commercially available kit (Diaclone, France) according to manufacture's introduction. The peptide pools covering the entire N protein of the Urbani strain were used to stimulate patients' peripheral blood mononuclear cells. The peptide pools were made of 57 peptides which are 15–18 amino acid residues in length overlapping by 10 amino acid residues. These peptides were obtained through the Biodefense and Emerging Infections Resources Repository at National Institute of Health (NIH) in the United States (http://www.beiresources.org/listing/index.cfm).

### Statistic analysis

Student's *t* test analysis was used to determine the significance of the results. Values of *p*≤0.05 indicated statistical significance.
